# Quantitative reconstruction of leukocyte subsets using DNA methylation

**DOI:** 10.1186/gb-2014-15-3-r50

**Published:** 2014-03-05

**Authors:** William P Accomando, John K Wiencke, E Andres Houseman, Heather H Nelson, Karl T Kelsey

**Affiliations:** 1 Department of Pathology and Laboratory Medicine, Brown University, Providence, RI 02912, USA; 2 Department of Neurological Surgery, University of California San Francisco, San Francisco, CA 94158, USA; 3 Department of Public Health, Oregon State University, Corvallis, OR 97331, USA; 4 Department of Epidemiology, University of Minnesota, Minneapolis, MN 55455, USA; 5 Department of Epidemiology, Brown University, Providence, RI 02912, USA

## Abstract

**Background:**

Cell lineage-specific DNA methylation patterns distinguish normal human leukocyte subsets and can be used to detect and quantify these subsets in peripheral blood. We have developed an approach that uses DNA methylation to simultaneously quantify multiple leukocyte subsets, enabling investigation of immune modulations in virtually any blood sample including archived samples previously precluded from such analysis. Here we assess the performance characteristics and validity of this approach.

**Results:**

Using Illumina Infinium HumanMethylation27 and VeraCode GoldenGate Methylation Assay microarrays, we measure DNA methylation in leukocyte subsets purified from human whole blood and identify cell lineage-specific DNA methylation signatures that distinguish human T cells, B cells, NK cells, monocytes, eosinophils, basophils and neutrophils. We employ a bioinformatics-based approach to quantify these cell types in complex mixtures, including whole blood, using DNA methylation at as few as 20 CpG loci. A reconstruction experiment confirms that the approach could accurately measure the composition of mixtures of human blood leukocyte subsets. Applying the DNA methylation-based approach to quantify the cellular components of human whole blood, we verify its accuracy by direct comparison to gold standard immune quantification methods that utilize physical, optical and proteomic characteristics of the cells. We also demonstrate that the approach is not affected by storage of blood samples, even under conditions prohibiting the use of gold standard methods.

**Conclusions:**

Cell mixture distributions within peripheral blood can be assessed accurately and reliably using DNA methylation. Thus, precise immune cell differential estimates can be reconstructed using only DNA rather than whole cells.

## Background

Different human cell types, defined by function and morphology, are enumerated in complex mixtures using a variety of physical, optical and proteomic characteristics [1]. Recent work shows that lineage-specific DNA methylation can also be used to distinguish different types of cells [2-7]. Patterns of DNA methylation, occurring at cytosine residues in the context of cytosine-guanine (CpG) dinucleotides, are tightly associated with chromatin conformation, which coordinates gene expression and reflects transcriptional programming of genes [8,9]. During differentiation, somatic cell lineages undergo *de novo* DNA methylation followed by maintenance methylation [10], thereby establishing mitotically heritable, cell lineage-specific methylation signatures [11-14]. As a result, patterns of DNA methylation serve as reliable indicators of cell lineage and can be used as sensitive and specific biomarkers for diverse cell types [2-6,13,15,16]. This suggests that DNA methylation can be used to simultaneously quantify multiple cell types in complex mixtures, but more extensive validation is required before this approach can be considered a viable alternative to established methods.

The immune system is a powerful model for investigating, developing and implementing new approaches to human cell detection and quantification. Blood is a complex mixture of many different specialized cell types and the composition of white blood cell (WBC, or leukocyte), populations is well known to reflect disease states and toxicant exposures [17-23]. Thus, the ability to detect an improper balance of immune cells is valuable in both a clinical and research setting. However, research aimed at further understanding immune cell level alterations is restricted by the limitations of immunodiagnostic methods. Routine blood leukocyte identification is achieved using physical cell isolation and the electrical impedance or optical light scattering properties of the cells [24]. Fluorescently labeled antibodies and flow cytometry are used to identify specialized cell subtypes, detecting proteins expressed at the cell membrane (for example, CD4+ T cells [25]). These methods rely upon intact cells, and therefore require fresh samples and cannot be applied to older, archived blood samples. Of course, it is now recognized that the expression of these cellular surface protein markers is controlled epigenetically. In fact, normal leukocyte lineage-specific differentiation is directed by differences in gene expression associated with distinct patterns of DNA methylation, with differentially methylated regions (DMRs) delineating distinct leukocyte subtypes [3-6,11,12,26].

We previously developed mathematical principles allowing for simultaneous quantification of multiple different immune cell subtypes in human blood using a reference dataset consisting of DMRs for purified WBC subsets [27]. That work quantified WBC subsets in simple mixtures, and used publicly available DNA methylation data to illustrate the feasibility of the approach. Here, we identified additional reference DMRs used to assess expanded numbers of leukocyte subsets, validated the approach with multiple platforms, quantified WBC subsets in complex mixtures emulating human blood, and compared the method with the current gold standards for immune profiling using human whole blood samples.

## Results and discussion

### Cell differential quantification using DNA methylation

Our goal was to use DNA methylation in order to detect and quantify the proportions of human T cells, B cells, natural killer (NK) cells, monocytes, basophils, eosinophils and neutrophils in any single blood sample. The first step in achieving this goal was to establish a reference library of DNA methylation signatures that will serve as biomarkers for those cell types. This was accomplished by using a microarray to assess DNA methylation in WBC subsets that were purified from normal (disease-free) human blood, thereby generating a reference dataset. To generate a target dataset, DNA methylation at the same CpG loci as the reference data set must be assessed in the target samples using the same platform that was used to establish the reference library. Then, the aforementioned cell types of interest are quantified in the target samples by projecting their DNA methylation profiles onto the mean methylation profiles for the purified WBC types of interest from the reference dataset using quadratic programming, as previously described [27]. Sample workflows are illustrated in Figure S1 in Additional file 1.

### DNA methylation distinguishes white blood cell subsets

We collected venous whole blood from 79 disease-free human donors (Table 1) and isolated homogenous populations of the WBC types of interest using magnetic activated cell separation (MACS) with the purity confirmed by fluorescence activated cell sorting (FACS; Figure S2 in Additional file 1). To minimize the impact of inter-individual variation in the selection of loci, at least four samples of each cell type were purified from different donors with varied demographics, including age, gender and ethnicity (Figure S3 in Additional file 1; Table 1). To identify patterns of WBC lineage-specific DNA methylation, a subset of these purified cell samples were run on a high-density methylation microarray (HDMA), the Infinium HumanMethylation27 (Illumina Inc., San Diego, CA, USA), which assessed DNA methylation at 27,578 CpG loci in 14,495 genes throughout the human genome. Applying a linear mixed effects model to these data (with cell type as the fixed effect and beadchip as the random effect) revealed hundreds of CpG loci exhibiting lineage-specific DNA methylation patterns that distinguish the WBC types of interest (Additional file 2).

**Table 1:** Demographic characteristics of blood donors for purified cells

Total, number	79
Age, mean (SD)	30 (9)
Weight (lbs), mean (SD)	181 (38)
Height (inches), mean (SD)	69 (3.7)
Gender	
Male, number (%)	62 (78%)
Female, number (%)	15 (19%)
Unknown, number (%)	2 (3%)
Race	
White, number (%)	32 (41%)
Hispanic, number (%)	12 (15%)
Black, number (%)	13 (16%)
Asian, number (%)	13 (16%)
Native American, number (%)	3 (4%)
Unknown/other, number (%)	6 (8%)
Tobacco smoking	
Yes, number (%)	13 (16%)
No, number (%)	33 (42%)
Unknown, number (%)	33 (42%)

**Figure 1:**
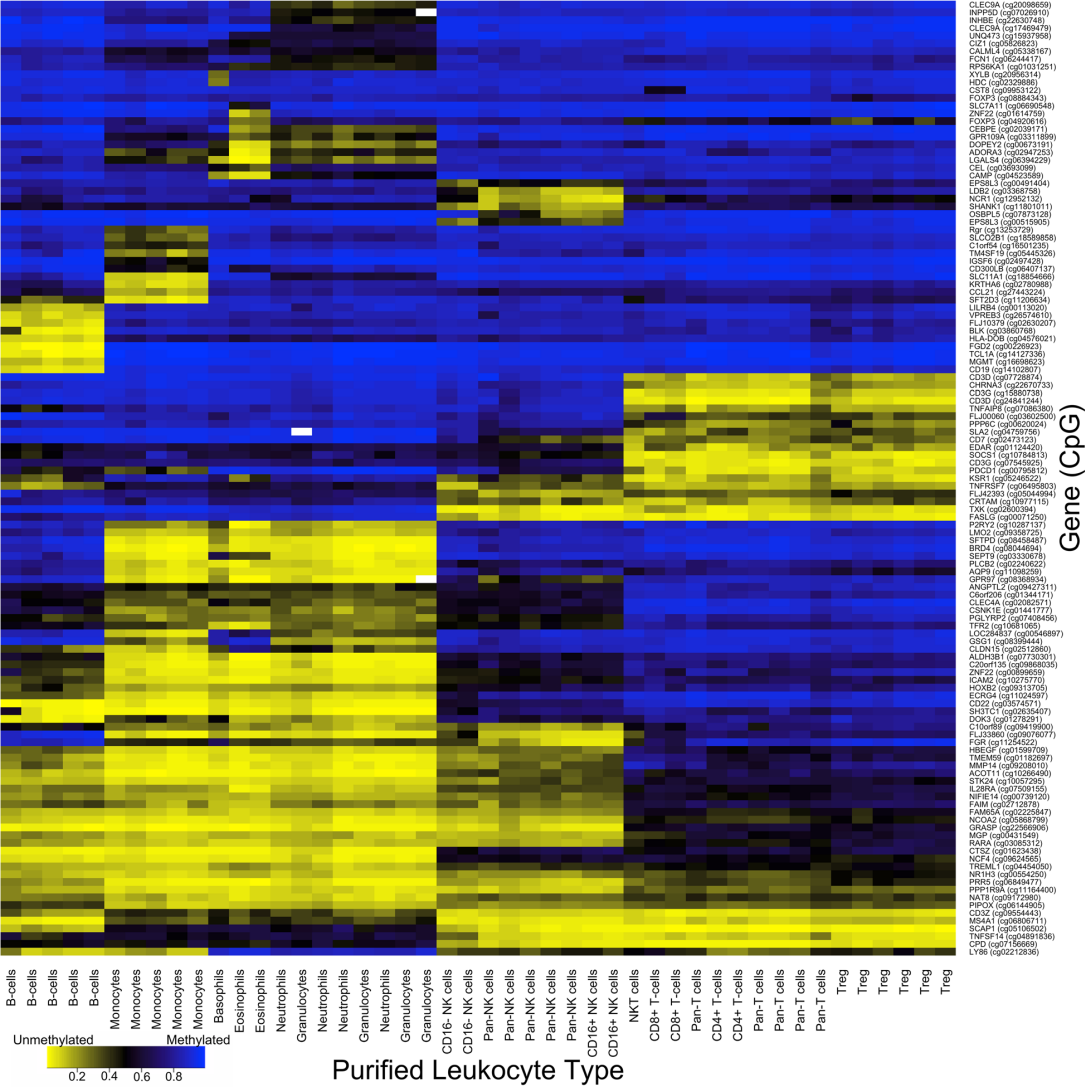
**DNA methylation signatures distinguishing normal human leukocyte subtypes on a high-density DNA methylation microarray.** Purified WBC subset samples are displayed in columns with cell type indicated at the bottom on the x-axis. Individual CpG loci are displayed in rows with the gene containing each locus indicated to the right on the y-axis. Methylation values range from completely unmethylated (yellow) to completely methylated (blue) as indicated in the key at the bottom left. Samples and loci are organized according to unsupervised, hierarchical clustering.

**Figure 2:**
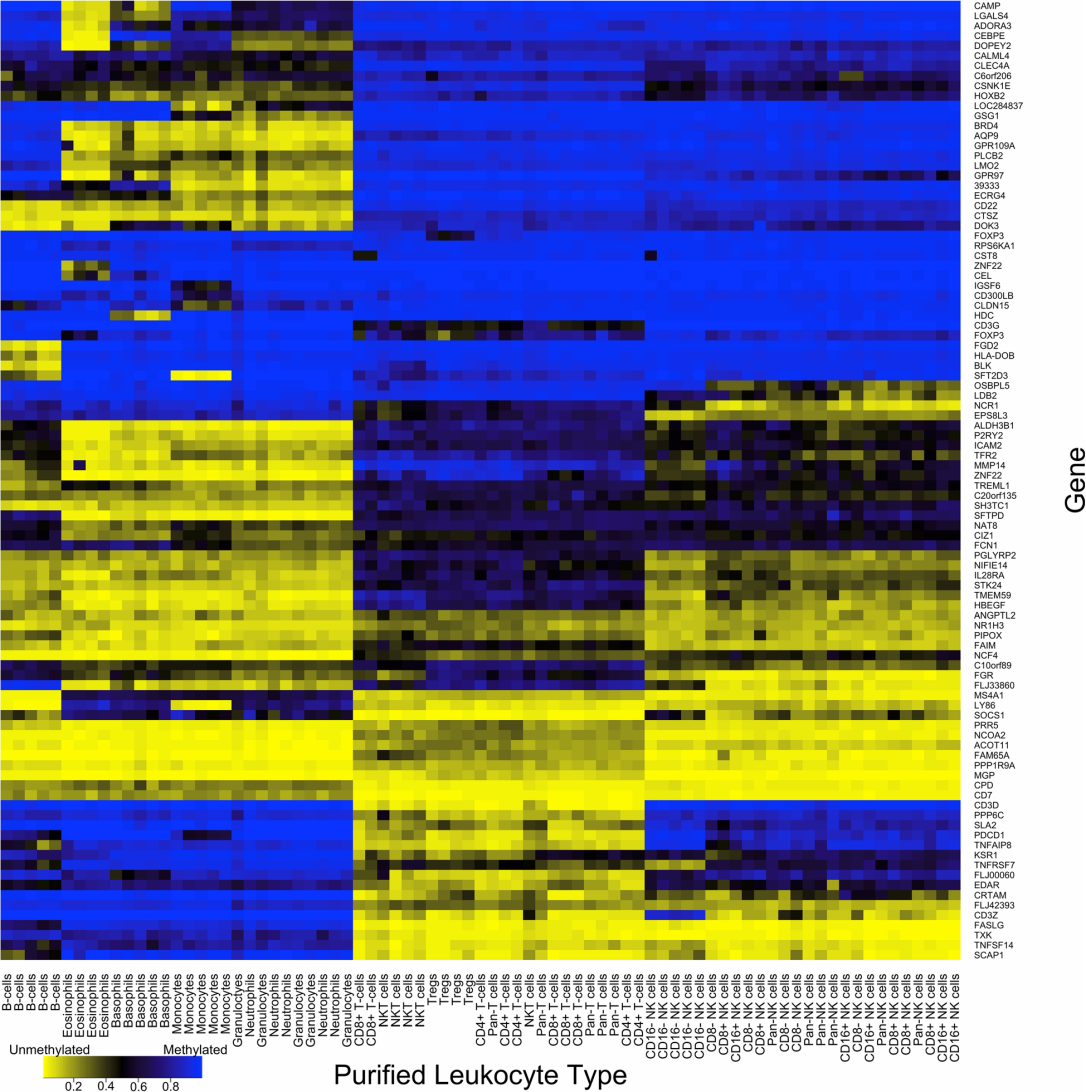
**DNA methylation signatures distinguishing normal human leukocyte subtypes on a custom, low-density DNA methylation microarray.** Purified WBC subset samples are displayed in columns with cell type indicated at the bottom on the x-axis. Individual CpG loci are displayed in rows with the gene containing each locus indicated to the right on the y-axis. Methylation values range from completely unmethylated (yellow) to completely methylated (blue) as indicated in the key at the bottom left. Samples and loci are organized according to unsupervised, hierarchical clustering.

We wanted to select a panel of 96 CpG loci that work well in concert for DNA methylation-based immune profiling, so that we could place these loci on a custom low-density DNA methylation microarray (LDMA), the VeraCode GoldenGate Methylation Assay microarray (Illumina Inc.), which would allow independent confirmation of the HDMA results, and would lead to more efficient use of resources for the quantification of WBC subsets in target samples. Therefore, we applied a bioinformatic search algorithm that works in a stochastic manner, substituting CpG loci and assessing the predictive ability of the selected loci by analyzing the variance in methylation across WBC types as designated in a contrast matrix. A panel of 96 CpG loci were selected where DNA methylation clearly distinguishes all of the WBC types of interest, including B cells, T cells, NK cells, monocytes, neutrophils, basophils and eosinophils, as indicated by unsupervised hierarchical clustering of HDMA data for the purified WBC subsets (Figure 1; Additional file 2). These 96 CpG loci were placed on the LDMA, which uses different chemistry than the HDMA and therefore represents an independent platform. Unsupervised hierarchical clustering of LDMA data for the purified WBC subsets confirmed that DNA methylation at these loci clearly and reliably distinguishes all of the WBC types of interest (Figure 2).

### Accurate prediction of purified WBC subset identities using DNA methylation

To optimize the performance of the method using a minimum number of CpG loci, we treated each (HDMA and LDMA) dataset consisting of purified WBC DNA methylation profiles as a target dataset containing unknown samples. Projection was performed using quadratic programming to estimate the proportions of seven different leukocyte subtypes, as continuous values ranging from 0% to 100%, in each of the purified WBC subset samples using methylation signatures from the corresponding HDMA or LDMA reference library. This 'crosscheck' procedure allowed us to optimize the method and improve efficiency by identifying any problematic purified WBC subset samples in the reference set, and determining the minimum number of CpG loci required for accurate leukocyte subtype detection and quantification. We found that only 34 CpG loci on the HDMA and 20 CpG loci on the LDMA were required to accurately predict the identity of purified WBC subset samples, as indicated by estimated proportions of the correct cell types very close to 100%, and estimated proportions of the incorrect cell types very close to 0% (Figures S4 and S5 in Additional file 1). For comparison, estimated WBC subtype proportions are also shown in Figures S4 and S5 in Additional file 1 for mixtures of WBC DNA in proportions found in human blood, and for human whole blood samples. Permutation tests (10,000 permutations) indicated that the observed success rates in prediction of purified WBC identities were significantly unlikely to occur by chance (*P* < 0.0001 for both platforms). The minimum sets of gene regions employed on each platform are indicated in Additional file 2.

The disparity in the minimum number of loci required on the two platforms is explained by the fact that fewer purified WBC subset samples were run on the HDMA (due to higher costs associated with that platform) and more CpG loci were therefore needed to compensate. In addition, this procedure revealed that CD16^-^CD56^bright^ 'regulatory' NK cells should be eliminated from subsequent reference data sets, since this cell type was frequently misclassified. These cells are not present in significant numbers in the peripheral blood; they are primarily found in lymphatic tissue. The purities of the regulatory NK cell samples obtained from peripheral blood were low according to FACS analysis (Figure S2I in Additional file 1), providing one plausible explanation for their consistent misclassification.

### Accurate quantitative reconstruction of WBC subsets using DNA methylation

**Figure 3:**
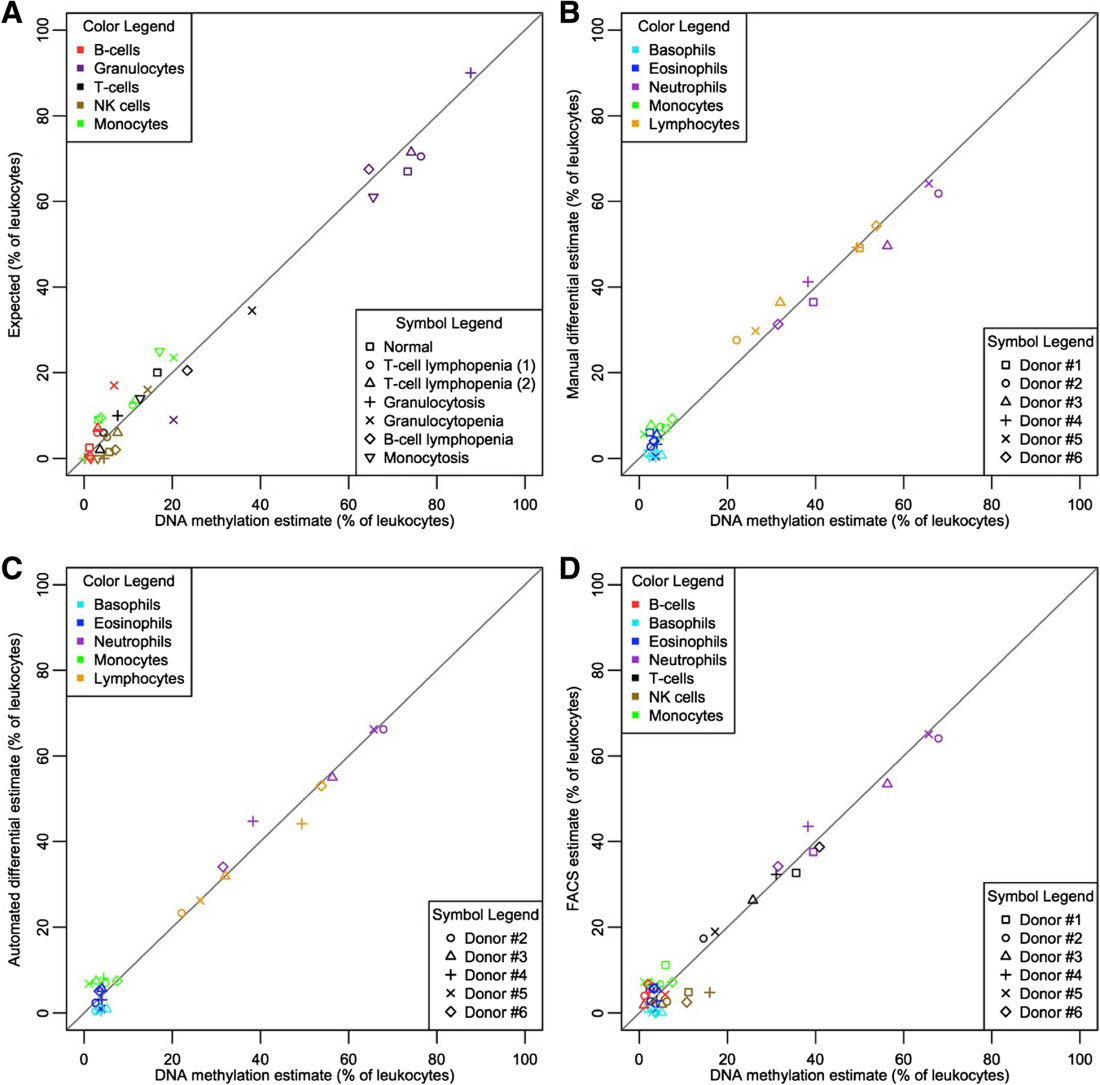
**Quantitative reconstruction of leukocyte subsets using a custom, low density DNA methylation microarray.** In all panels, the x-axis indicates the quantities of specific WBC subsets determined using DNA methylation. Cell type is indicated by color and sample type is indicated by shape of the point, as described in the inset legends. Lines are drawn from the origin with a slope of one indicating ideal correspondence between the displayed values in each panel. **(A)** DNA from purified WBC subsets was combined in quantities mimicking human blood under clinical conditions. The expected quantity of each cell type is indicated by the y-axis. **(B-D)** Whole blood samples from disease-free human donors subjected to WBC subset quantification by established methods. Results of the established methods are shown on the y-axis and include manual 5-part differential **(B)**, automated 5-part differential **(C)** and FACS **(D)**.

We next sought to determine the efficacy of our method for determining the range of peripheral blood leukocyte populations in commonly encountered human health conditions. In order to accomplish this, genomic DNA extracted from five of the purified WBC subset samples was combined in precise quantities that mimicked human blood found in patients exhibiting specific clinical conditions, as well as in quantities emulating 'normal' human blood in a typical individual (Table 2). We then assessed DNA methylation in these DNA mixtures using both the HDMA and LDMA platforms, and quantified the five different WBC types in each mixture by performing our projection via quadratic programming using the appropriate reference data set, utilizing only the minimum numbers of CpG loci established by the crosscheck procedure described above (34 or 20 loci). In all of these samples on both platforms, the five WBC quantities measured using DNA methylation were very close to the expected values (Figure S6A in Additional file 1; Figure 3A), providing strong evidence that this approach is capable of determining the composition of leukocytes in human peripheral blood.

**Table 2:** Proportions of DNA from purified cells combined into mixtures that artificially reconstruct blood under clinical conditions

**Clinical condition**	**T cells**	**B cells**	**NK cells**	**Granulocytes**	**Monocytes**
Normal	20%	2.5%	1.5%	67%	9%
T-cell lymphopenia 1	6%	6%	5%	70.5%	12.5%
T-cell lymphopenia 2	2%	7%	6%	71.5%	13.5%
Granulocytosis	10%	0%	0%	90%	0%
Granulocytopenia	34.5%	17%	16%	9%	23.5%
B-cell lymphopenia	20.5%	0.5%	2%	67.5%	9.5%
Monocytosis	14%	0%	0%	61%	25%

Next, we directly compared our approach to gold standard methods of WBC quantification that are routinely applied to human peripheral blood. Venous whole blood was collected from six different, disease-free, human donors (Additional file 3) and was immediately subjected to three different, well established immune profiling methods: manual 5-part differential, complete blood count (CBC) with automated 5-part differential, and FACS. Genomic DNA was extracted from these six blood samples, and DNA methylation was assessed using both the HDMA and LDMA platforms. The appropriate WBC types were quantified in each sample using DNA methylation by projection via quadratic programming using the corresponding reference data set, utilizing only the minimum numbers of CpG loci established by the crosscheck procedure described above (34 or 20 loci). For all samples on both platforms, the quantities of WBC types of interest measured by DNA methylation were highly correlated with the established, gold standard methods of WBC quantification (Figure 3B-D; Figure S6B-D in Additional file 1). In fact, these results were similar to those observed when comparing the three traditional methods to each other (Figure S7 in Additional file 1).

To consider the differences between the 'expected' values (that is, known WBC DNA amounts in the mixtures, or measurements obtained using gold standard methods) and the 'observed' values (that is, estimates obtained using DNA methylation), we constructed Bland-Altman plots showing the difference between each pair of values relative to the mean of these two values. We calculated the root-mean-square-error (RMSE) for each pair of values that indicates the standard deviation in model prediction error. This analysis was performed for each pair of gold standard measurements. These results indicate that the agreement between our approach and each of the gold standard methods was excellent, and we observed little evidence of systematic bias. The mean differences between expected and observed values were near zero, and RMSE values were generally around 3 to 4.3 percentage points (Figure S8 in Additional file 1). These levels of uncertainty were similar to those seen when agreement and RMSE were assessed between the gold standard methods, though FACS and automated differential values showed slightly better agreement than the others with a RMSE of about two percentage points (Figure S9 in Additional file 1).

Interestingly, when optimized independently, the best minimum sets of CpG loci were different on the two platforms examined. There are several explanations for this fact. First, a greater number of purified WBC reference samples were run in experiments using the LDMA. Second, the chemistries of the two platforms were sufficiently different that not all loci performed exactly the same on the LDMA (GoldenGate technology) as they did on the HDMA (Infinium technology). In fact, some very meaningful CpG loci that were on the HDMA could not be placed on the custom LDMA due to limitations of the technology, and in some instances we used nearby CpGs instead (for example, the *CD3Z* promoter). Thus, some CpG loci that were excluded from the final analyses are still biologically meaningful, but since DNA methylation patterns at multiple CpG loci can signal the same phenotype, not all of these loci are needed to obtain accurate estimates of cell mixture proportions. Thus, different platform-specific optimal sets of cell lineage-specific DMRs reflect the same biology. This suggests the best panel of gene regions for a given platform should be identified by a mixed strategy using mathematics combined with curation based on biological knowledge, and considering the chemistries of different platforms. Third, even gold standard methods show differences in measurements obtained using different platforms, so it is not surprising to find some differences between independent methylation platforms.

### Storage conditions do not affect WBC estimates obtained using DNA methylation

**Figure 4:**
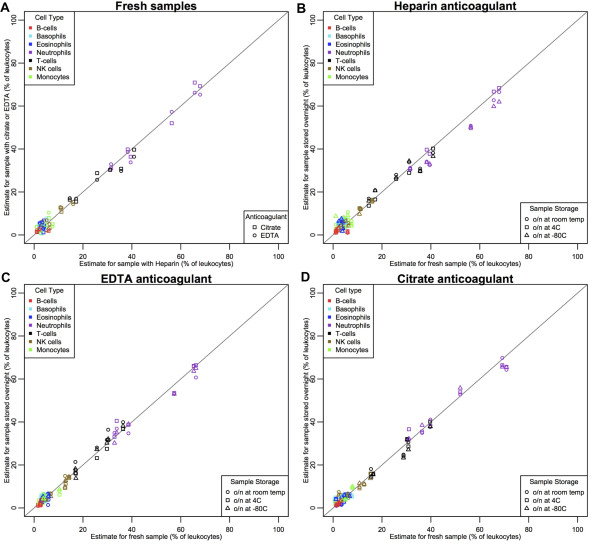
**Comparisons of DNA methylation-based immune cell quantification (using the LDMA) for different blood anticoagulants and storage conditions.** All blood samples were from disease-free human donors. Lines are drawn from the origin with a slope of one indicating ideal correspondence between the displayed values in each panel. In all panels, cell type is indicated by color and shape of the point indicates **(A)** blood anticoagulant or **(B-D)** storage condition in the same blood anticoagulant, including heparin **(B)**, EDTA **(C)**, and citrate **(D)**.

The stability of DNA suggests that our method can overcome many limitations of previous WBC quantification methods, since this novel approach does not require fresh blood or an intact cell membrane. This implies that our method should be applicable to samples that were previously precluded from immunological assessment, such as archived blood samples that have been stored in hospitals and laboratories, or blood samples that were collected in an anticoagulant that is not compatible with a particular method. We sought to verify that blood anticoagulant and storage temperatures do not alter WBC quantification by DNA methylation. Each of the six venous whole blood samples from disease-free human donors was collected into three different anticoagulants: citrate, heparin and EDTA. A portion of each of these samples was used for DNA extraction (that is, using fresh blood) and the remainder was then divided into three aliquots that were stored at room temperature, 4°C or -80°C for at least 24 hours prior to DNA extraction (Figure S10 in Additional file 1). All of these DNA samples were then run on the LDMA platform to assess DNA methylation, generating a target data set to consider the effects of blood storage conditions. All seven WBC types of interest were quantified in each of these target samples by performing our projection via quadratic programming using the LDMA reference set, utilizing only the minimum number of 20 CpG loci established by the crosscheck procedure described above. Results indicated that the storage conditions examined did not alter WBC subset quantities measured in human blood by DNA methylation (Figure 4).

## Conclusions

Because our DNA methylation-based approach is robust across platforms, highly correlated with widely utilized gold standard methods and independent of blood collection and storage conditions, it will enable immune profiling to be performed in a wide variety of samples that were previously precluded from immunological assessment. Such profiling includes archived blood samples from large epidemiologic studies allowing investigators to account for shifts in normal immune cell distributions [28,29].

The current work is sufficient to validate the method for these research applications and it indicates that the method’s dynamic range of sensitivity is within a reasonable scale to warrant further consideration as having clinical utility. This method may serve as a reliable alternative to the accepted reference standard of manual differential, as well as the automated differential, and even FACS-based analysis. It will be necessary to confirm that lineage-specific DMRs are consistent across ages, ethnicities and genders in these applications. However, recently Koestler *et al*. [30] reported that DNA methylation-based prediction of leukocyte subset profiles was not biased by demographic factors. This suggests that fundamental leukocyte epigenetic programming is consistent between individuals with different demographic characteristics.

Looking ahead we envision that DNA methylation-based approaches might be used to differentiate and enumerate any type of lineage-stable human cells within complex mixtures. This presents an unprecedented opportunity for the development of a new generation of methods for cellular quantification that exploits the human methylome; supporting the feasibility of 'molecular' histology. Using the immune system as a model, we have created a paradigm for the mapping of cell-specific DNA methylation signatures in order to generate reference libraries of efficacious biomarkers that distinguish different cell types. Moreover, we have established powerful computational tools to quantitatively reconstruct the precise makeup of cellular mixtures. In the past, simultaneous quantification of normal or disease-associated changes in cell population composition has been accomplished using flow cytometry, electrical impedance, light scatter and/or immunohistochemistry. This can require large volumes of fresh blood or tissue, and, for flow cytometry, can involve laborious antibody tagging [31,32]. In contrast, our approach is high-throughput and entails simple, convenient DNA analysis techniques that can easily be automated to facilitate rapid quantitative reconstruction of cell subsets.

## Materials and methods

Figure S1 in Additional file 1 summarizes the workflow carried out for all samples, derived from human whole blood, that were utilized in this work. Blood was obtained through an institutional review board-approved donor program at AllCells, LLC (Alameda, CA, USA) and research was carried out in accordance with the Helsinki declaration. Samples were de-identified and randomized prior to sodium bisulfite conversion and subsequent DNA methylation interrogation; thus, researchers were blind to specific sample identities. Data for all DNA methylation microarray experiments are available on NCBI’s Gene Expression Omnibus (GEO), in accordance with MIAME, under accession numbers GSE39981 (HDMA for purified leukocyte subtypes), GSE54647 (LDMA for purified leukocyte subtypes, artificial bloods, and whole bloods) and GSE54670 (HDMA for artificial bloods and whole bloods).

### Purified leukocyte subtypes

Venous whole blood samples were collected from 79 disease-free human donors whose demographic characteristics are shown in Table 1. Each of these samples was used to obtain a homogenous population of one specific type of leukocyte, which was purified by MACS, a method of cell separation that utilizes antibody-conjugated magnetic microbeads, using a combination of positive and negative selection protocols (Miltenyi Biotec Inc., Auburn, CA, USA). All 79 purified cell samples had their purity confirmed by FACS. Representative FACS results for all 15 of the sample types are shown in Figure S2 in Additional file 1. The hierarchical relationship between all of the different populations of MACS purified leukocyte subtypes, as well as the number of replicate samples for each cell type, is illustrated in Figure S3 in Additional file 1.

### Conventionally profiled whole bloods

Six additional venous whole blood samples were collected from different disease-free human donors whose demographic characteristics are summarized in Additional file 3. The workflow for these samples is illustrated in Figure S10 in Additional file 1. Each of the six whole blood samples was first divided into three aliquots, each containing a different anticoagulant: heparin, citrate or EDTA. For each blood sample, portions of the aliquot in heparin were used to perform conventional immune profiling methods, including flow cytometry (described below), manual 5-part white blood cell differential and CBC with automated 5-part white blood cell differential. Another portion of this aliquot for each sample was subjected to methylation assessment on the HDMA (described in detail below). A portion of all three aliquots for each blood sample was also subjected to methylation assessment on the LDMA (described in detail below) without being stored overnight. The remainder of these three aliquots for each of the six blood samples was divided into three more aliquots, each to be stored overnight at a different temperature (room temperature, 4°C, and -80°C) prior to methylation assessment on the HDMA.

### Differential leukocyte counts

Manual WBC counts were performed according to established standards [33,34] and automated WBC counts were performed using the XE-5000™ Automated Hematology System (Sysmex America, Inc., Mundelein, IL, USA) according to the manufacturer's instructions. Cell types that were enumerated included total WBC, lymphocytes, monocytes, neutrophils, basophils and eosinophils.

### Fluorescence activated cell sorting of leukocyte subsets

Blood samples were directly stained for cell surface markers, and incubated for 20 minutes in the dark at 4°C. All antibodies used were purchased from eBioscience, Inc. (San Diego, CA, USA). Each blood sample was split into two aliquots. In the first aliquot cells were stained with: anti-human CD3e FITC (catalog number 11-0039-41), anti-human CD4 APC-eFluor 780 (catalog number 47-0049-41), anti-human CD8a 605NC (catalog number 93-0088-41), anti-human CD16 PE-Cy7 (catalog number 25-0168-41), anti-human CD25 APC (catalog number 17-0259-41), anti-human CD45 PerCP-Cy5.5 (catalog number 45-9459-41), anti-human CD56 PE (catalog number 12-0567-41), and anti-human CD127 eFluor 127 (catalog number 48-1278-41) to analyze T cells, NKT cells, and NK cells. In the second aliquot, cells were stained with: anti-human CD14 FITC (catalog number 11-0149-41), anti-human CD15 eFluor 450 (catalog number 48-0159-41), anti-human CD16 PE-Cy-7, anti-human CD19 APC-eFluor 780 (catalog number 47-0199-41), anti-human CD45 PerCP-Cy5.5, and anti-human CD123 PE (catalog number 12-1239-41) to analyze B cells, monocytes, and granulocytes (neutrophils, eosinophils, and basophils).

Unstained, isotype, and fluorescence-minus-one (FMO) controls were used to determine sample gating and background. Individual compensation controls were used in each sample run. CountBright counting beads (Invitrogen, catalog number C36950) were added for cell quantification. Acquisition was performed within 12 hours of blood draw on the FACSAria III flow cytometer (Becton Dickinson, Franklin Lakes, NJ, USA) using FACSDiva Software (Becton Dickinson). An acquisition limit of 10,000 events was used on the monocyte gate, using a forward scatter (FSC) versus side scatter (SSC) dot plot, for each aliquot. Final data analysis and presentation of results was done using Flowjo software (TreeStar, Inc., Ashland, OR, USA).

The following outlines the cell types and detection parameters. Lymphocytes: low SSC and low FSC; B cells: CD45+ and CD19+; T cells: CD45+ and CD3+ antibodies; helper T cells (Th): CD3+ and CD4+; regulatory T cells (Tregs): CD3+ and CD4+ and CD25+ and FOXP3+; cytotoxic T cells (Tc): CD3+ and CD8+; NK T cells (NKT): CD3+ and C56+; NK cells: CD3- and CD56+; effector NK cells: CD3- and CD16+ and CD56 dim (that is, lower level); regulatory NK cells: CD3- and CD16- and CD56 bright (that is, higher level); CD8+ NK cells: CD3- and CD8+ and CD56+ antibodies; CD8- NK cells: CD3- and CD8- and CD56+; granulocytes: high SSC and high FSC; eosinophils: CD44+ and high SSC and high FSC; basophils: CD123+ and high SSC and high FSC; neutrophils: CD15+ and CD16+ and high SSC and high FSC; monocytes: low SSC and high FSC and CD14 + .

### DNA extraction

Genomic DNA was extracted and purified from whole blood and from MACS purified leukocyte samples using AllPrep DNA/RNA/Protein Mini Kit (QIAGEN, Valencia, CA, USA, catalog number 8004) or DNeasy blood and tissue kit (QIAGEN, Cat No. 69506) according to the manufacturer’s protocol. DNA was then quantified by NanoDrop ND-1000 Spectrophotometer (NanoDrop Technologies, Inc., Wilmington, DE, USA). Some samples were then further purified using the DNA Clean and Concentrator according to the manufacturer’s protocol (ZYMO Research Corp., Irvine, CA, USA, catalog number D4004). Samples were kept at 4°C for short-term storage or at -20°C for long-term storage.

### Artificial blood samples

Genomic DNA from five of the purified leukocyte samples was combined in quantities that mimicked human blood under seven clinical conditions (Table 2). DNA was mixed thoroughly and stored briefly at 4°C prior to analysis.

### Sodium bisulfite conversion

Genomic DNA from the six conventionally profiled whole blood samples, genomic DNA from the 79 purified leukocyte samples, and DNA mixtures in the seven artificial blood samples were first randomized, then treated with sodium bisulfite using ZYMO EZ-96 DNA Methylation Kit (ZYMO Research Corp., catalog number D5004), and then stored at -80°C until needed. This enables assessment of DNA methylation by converting unmethylated cytosine residues to uracil.

### High-density DNA methylation microarray

To explore patterns of cell lineage-specific DNA methylation and examine the viability of our mathematical models, we ran 46 of the purified leukocyte DNA samples on the Infinium® HumanMethylation27 Beadchip microarray and six of the artificial blood reconstruction samples (excluding T-cell lymphopenia 1), and the six conventionally profiled whole blood samples on the Infinium® HumanMethylation450 Beadchip microarray (Illumina Inc.). These platforms quantify the methylation status of 27,578 CpG loci, and 485,577 CpG loci, respectively. The ratio of fluorescent signals is computed from both alleles using the following equation:

$$\beta =\left(\max\left(M,0\right)\right)/\left(\left|U\right|+\left|M\right|\right)+100$$

The resultant β-value is a continuous variable ranging from 0 (unmethylated) to 1 (completely methylated) that represents the methylation at each CpG site and is used in subsequent statistical analyses. Data were assembled with the methylation module of GenomeStudio software without normalization (Illumina, Inc.) [35].

Following the crosscheck optimization procedure, a minimum number of 34 CpG loci were selected to establish DNA methylation signatures for the HDMA reference library. These loci were found in the following genes: *CLEC9A* (2 loci), *INPP5D*, *INHBE*, *UNQ473*, *SLC7A11*, *ZNF22*, *XYLB*, *HDC*, *RGR*, *SLCO2B1*, *C1orf54*, *TM4SF19*, *IGSF6*, *KRTHA6*, *CCL21*, *SLC11A1*, *FGD2*, *TCL1A*, *MGMT*, *CD19*, *LILRB4*, *VPREB3*, *FLJ10379*, *HLA-DOB*, *EPS8L3*, *SHANK1*, *CD3D* (2 loci), *CHRNA3*, *CD3G* (2 loci), *RARA*, *GRASP*.

### Low-density DNA methylation microarray

To thoroughly validate our DNA methylation-based approach to immune profiling, we ran all 79 purified leukocyte samples, all seven artificial blood reconstruction samples, and all 72 samples of the six conventionally profiled whole blood samples (each stored under 12 different conditions) on the VeraCode® custom GoldenGate® Methylation assay (GGMA). This assay uses a four-probe design to differentiate between methylated and unmethylated sequences for a custom panel of 96 different CpG loci. This process involves generation of DNA targets through allele-specific amplification using universal primers, and hybridization to a bead array at sites bearing complementary address sequences. The hybridized targets contain a fluorescent label denoting a methylated or unmethylated state for a given locus. Methylation status of each interrogated CpG site is calculated as the ratio of fluorescent signal from one allele relative to the sum of both methylated and unmethylated alleles, thereby generating a β-value ranging from 0 (unmethylated) to 1 (fully methylated). Several different control types ensure data quality. Each bead type is represented with an average 30-fold redundancy. Data were assembled with the methylation module of GenomeStudio software (Illumina, Inc.) without normalization.

Following the crosscheck optimization procedure, a minimum number of 20 CpG loci were selected to establish DNA methylation signatures for the LDMA reference library. These loci were found in the following genes: *FGD2*, *HLA-DOB*, *BLK*, *IGSF6*, *CLDN15*, *SFT2D3*, *ZNF22*, *CEL*, *HDC*, *GSG1*, *FCN1*, *OSBPL5*, *LDB2*, *NCR1*, *EPS8L3*, *CD3D*, *PPP6C*, *CD3G*, *TXK*, *FAIM.*

### Statistical methods

All statistical analyses were performed using the R statistical platform [36] and code is provided in Additional file 4.

#### Identification of cell lineage-specific methylation

To identify DNA methylation signatures that represent biomarkers of leukocyte subtypes, we applied a linear mixed effects (LME) model to the purified leukocyte HDMA data with cell type designated as the fixed effect and beadchip as the random effect (controlling for plate effects). This generated F-statistics for every CpG on the array indicating how well differential methylation at that locus distinguishes seven different leukocyte lineages: T cells, B cells, NK cells, monocytes, eosinophils, basophils, and neutrophils. This also generated seven coefficients for each CpG indicating directionality and intensity of differential methylation at that locus for the cell types.

#### Selection of CpG panel for immune profiling

Using the LME results, we implemented a stochastic search algorithm to determine the best combination of putative DMRs to use for the simultaneous assessment of T cells, B cells, NK cells, monocytes and granulocytes in a human blood sample. This algorithm assesses the predictive ability of a selected panel of CpG loci by analyzing the variance in methylation across cell types as designated in a contrast matrix. If substitution of a randomly selected locus for one of the loci in the panel improves the predictive ability, the substitution is accepted and the new locus replaces the old in the panel. We implemented this search algorithm for 50,000 iterations starting from 10 different random number seeds in three stages: first starting with the top 500 F-statistics, then the top 500 absolute effect sizes (based on the LME coefficients), and then the top 500 from the first two stages. The stochastic search algorithm was then implemented one more time, starting from the top 96 from the final stage above until the acceptance rate for substitutions definitively dropped to zero.

#### DNA methylation-based cell quantification

To estimate cell mixtures by DNA methylation marks, we employ a constrained projection, wherein a DNA methylation profile from a target profile is projected onto mean methylation profiles for isolated cell types, subject to the constraint that the projection values (estimated mixing weights) are greater than or equal to zero and sum to less than one. The mean values are obtained from a reference library of DNA methylation signatures, and the projection is implemented via quadratic programming [37,38]. Details of this algorithm have previously been described [27].

#### Significance, correlation and error

To determine the probability of obtaining the observed success rates in predicting purified WBC subtype identities by chance using the crosscheck procedure displayed in Figures S4 and S5 in Additional file 1, we used the broadly defined cell type categories (monocyte, eosinophil, basophile, neutrophil, T cell, B cell and NK cell) as the 'true' category label, and calculated the sum of the predicted fractions that were correctly categorized within these broad types. This was the observed test statistic. For each permutation we shuffled the labels with respect to the samples and calculated the same statistic to obtain the permutation (null) distribution for the same statistic. Pearson correlations were calculated between expected and observed (or a given pair of observed) measurements of WBC subtype quantities in the cell mixtures and the human whole blood samples. RMSE values were calculated using the hydroGOF package via the following formula: sqrt(Mean((Expected - Observed)^2^).

### Microarray data

Microarray data have been deposited on NCBI’s Gene Expression Omnibus in accordance with MIAME under accession numbers GSE39981, GSE54647 and GSE54670.

## Abbreviations

CBC: complete blood count; CpG: cytosine-guanine dinucleotide; DMR: differentially methylated region; FACS: fluorescence activated cell sorting; FSC: forward scatter; HDMA: high-density methylation microarray; LDMA: low-density methylation microarray; LME: linear mixed effects; MACS: magnetic activated cell separation; NK: natural killer; RMSE: root-mean-square-error; SSC: side scatter; WBC: white blood cell.

## Competing interests

WPA, JKW, EAH and KTK (Brown University and University of California San Francisco) have filed a relevant patent.

## Authors’ contributions

WPA participated in the design and coordination of the study, carried out laboratory work, performed statistical analyses, and drafted the manuscript. JKW conceived of the study, participated in its design and coordination, and helped to draft the manuscript. EAH participated in the design of the study, developed the mathematical approaches, and helped to draft the manuscript. HHN participated in the design of the study and helped to draft the manuscript. KTK conceived of the study, and participated in its design and coordination, and helped to draft the manuscript. All authors read and approved the final manuscript.

## Supplementary Material

Additional file 1 All supplementary figures referenced in this manuscript. Click here for file

Additional file 2: Table S1 Which shows the results of the LME model applied to purified WBC HDMA data for the 96 gene regions placed on the LDMA, along with other annotations for the gene regions, including which regions were selected for final analyses using each platform. Click here for file

Additional file 3: Table S2 Which displays the demographic characteristics of the six disease-free human donors who provided whole blood samples for comparison to established methods of cell quantification and consideration of blood storage conditions. Click here for file

Additional file 4 R code used to perform statistical analyses. Click here for file
