# Supplementary material for: Quantitative reconstruction of leukocyte subsets using DNA methylation
Source: Genome Biol. 2014 Mar 5;15(3):R50. doi: 10.1186/gb-2014-15-3-r50 (PMC4053693; doi:10.1186/gb-2014-15-3-r50)

**Supplementary figure legends for:**

Accomando WP, Wiencke JK, Houseman EA, Nelson HH, and Kelsey KT. Quantitative reconstruction of leukocyte subsets using DNA methylation.

***Additional file 1: Figure S1:*** Workflow carried out for all samples, derived from human whole blood, utilized in this work.


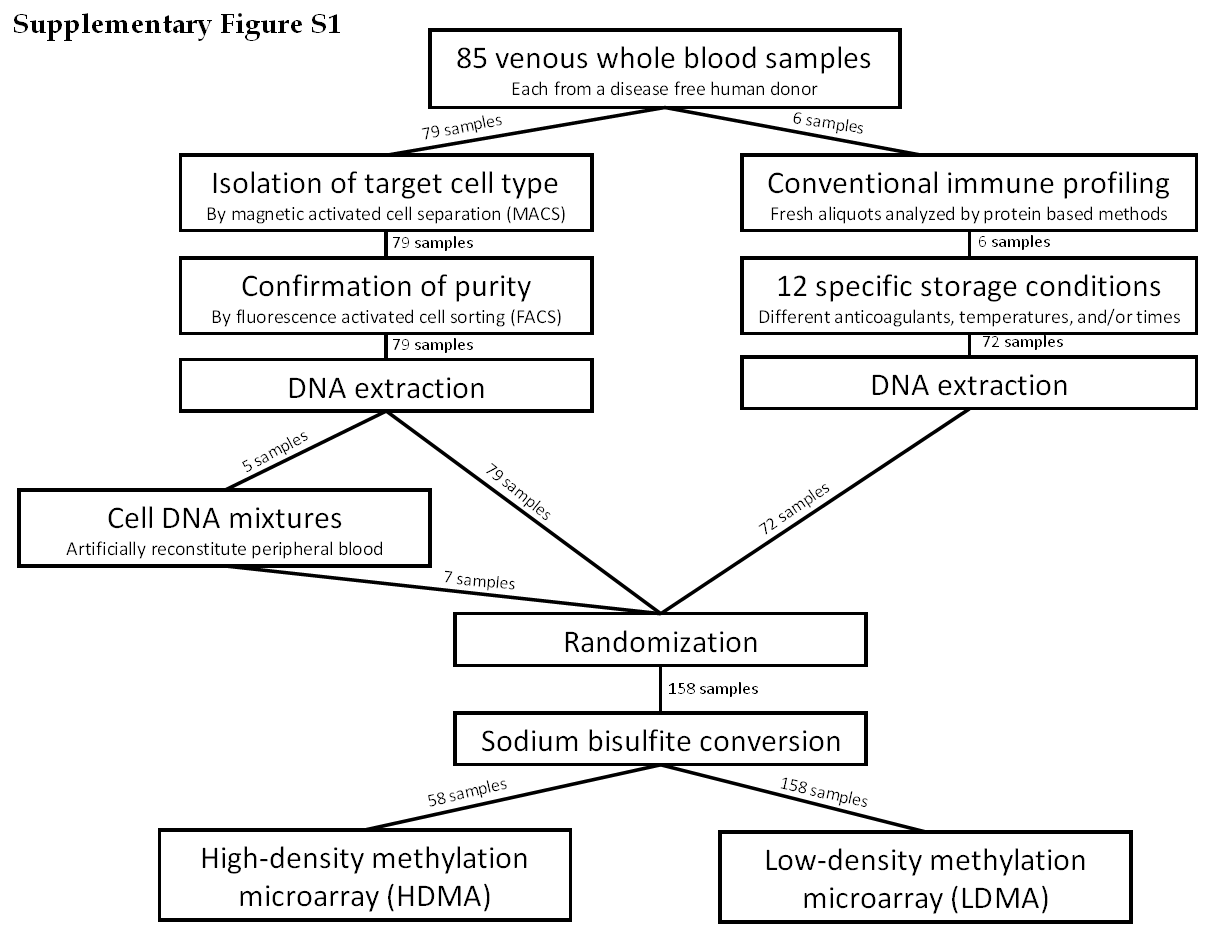


***Additional file 1: Figure S2:*** Representative FACS results for all types of purified WBC subsets used in this work. The lower right quadrant of each panel indicates sample purity. The upper right quadrant of each panel indicates the viability of the cells in the sample.


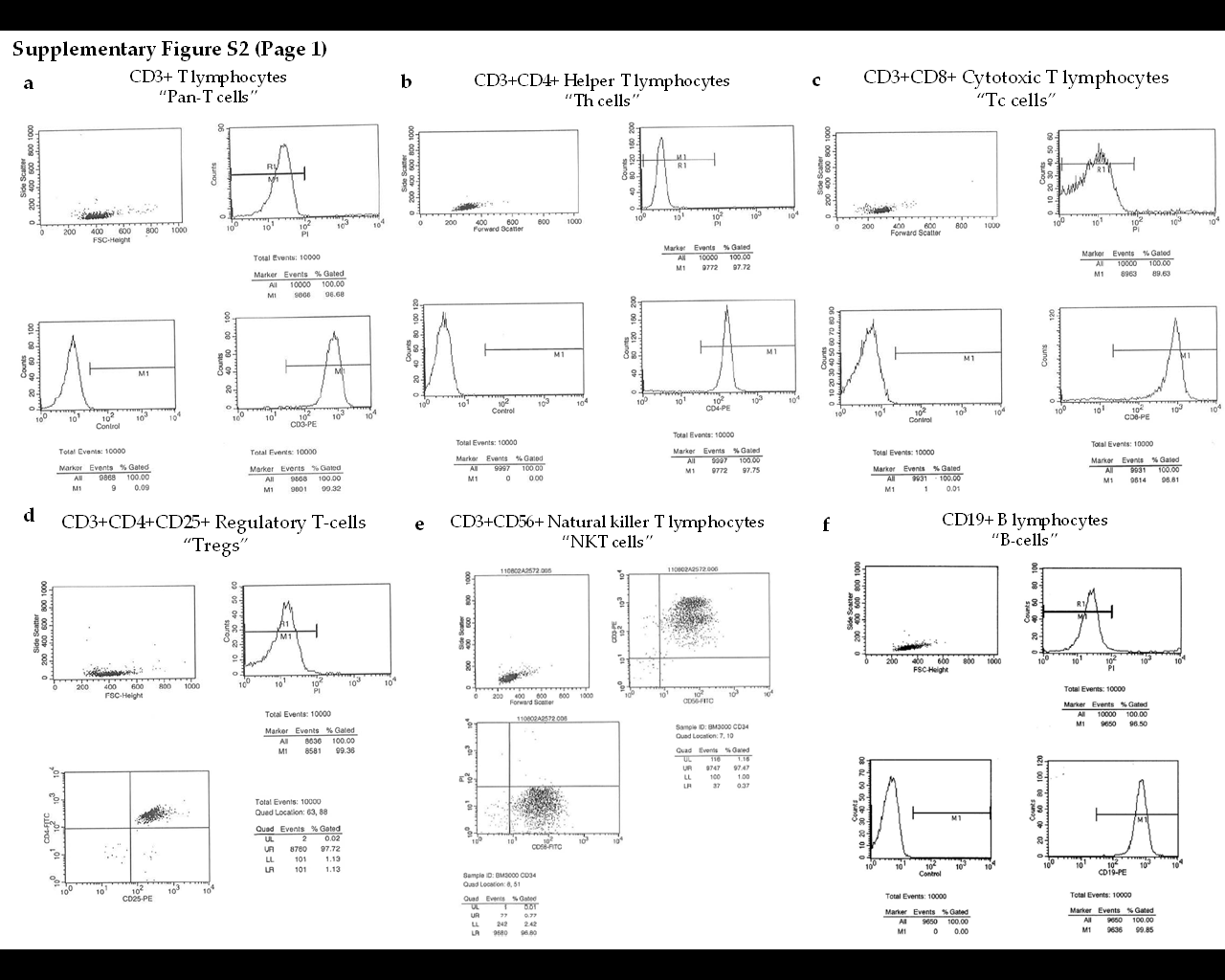


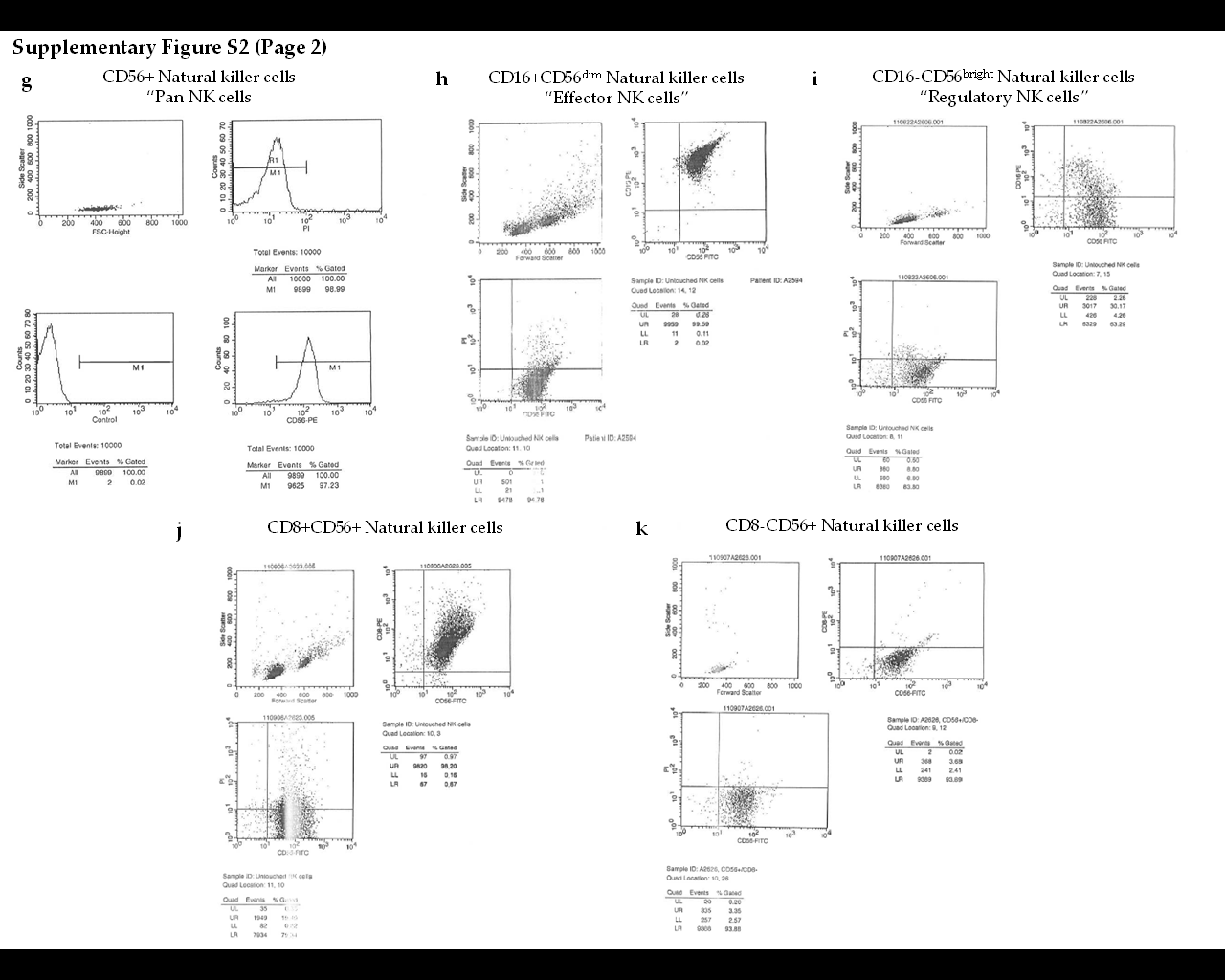


***
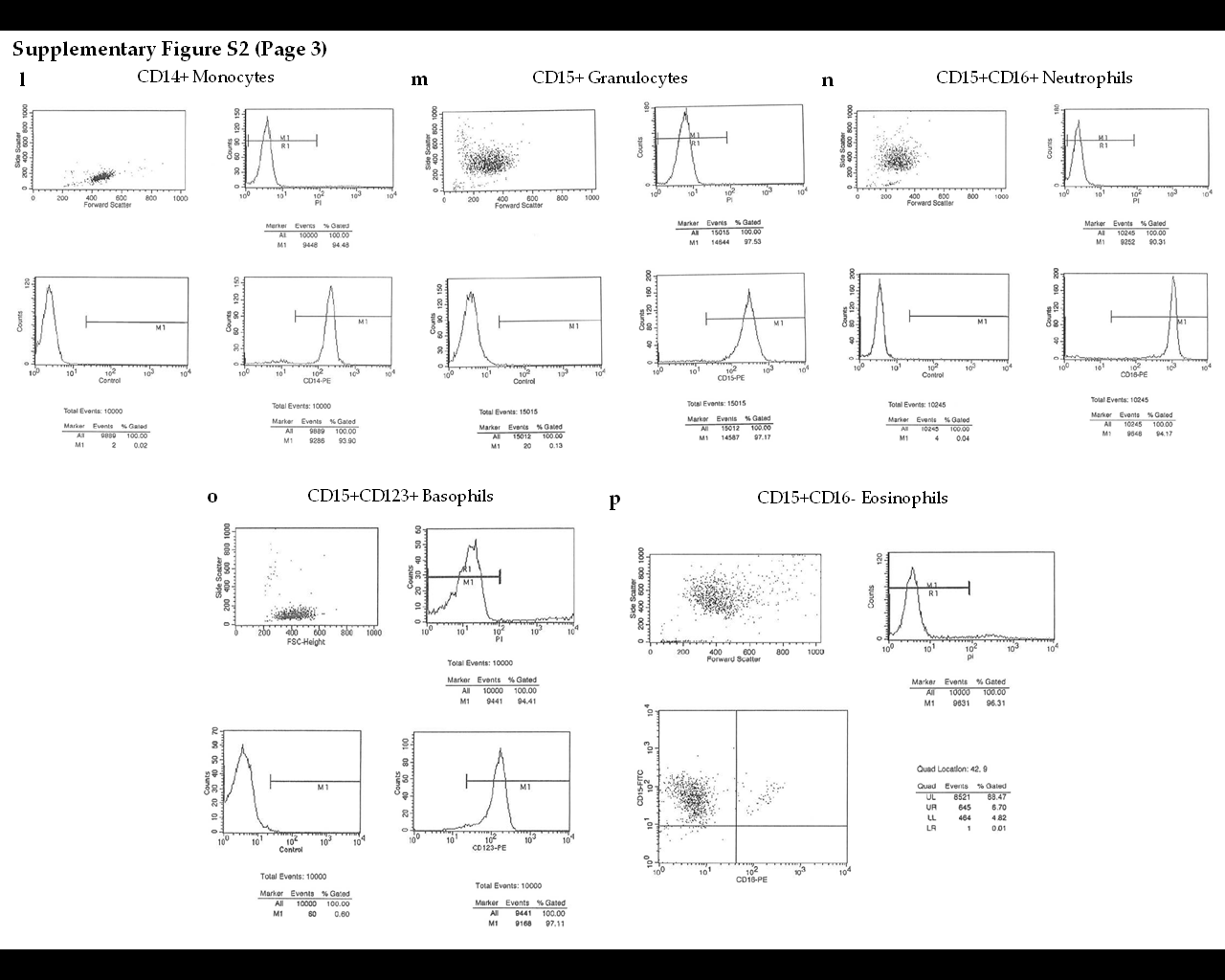
***

***Additional file 1: Figure S3:*** MACS purified WBC subset samples used to establish reference libraries of DNA methylation signatures. Terminal nodes represent the final sample cell types, which were each purified from a disease-free human blood specimen. The tree diagram indicates the hierarchical relationship of sample cell lineages. * Pan samples did not undergo any subsequent selection in the MACS separation process, and therefore contain a biological mixture of subsets within the cell type immediately above them in the tree.


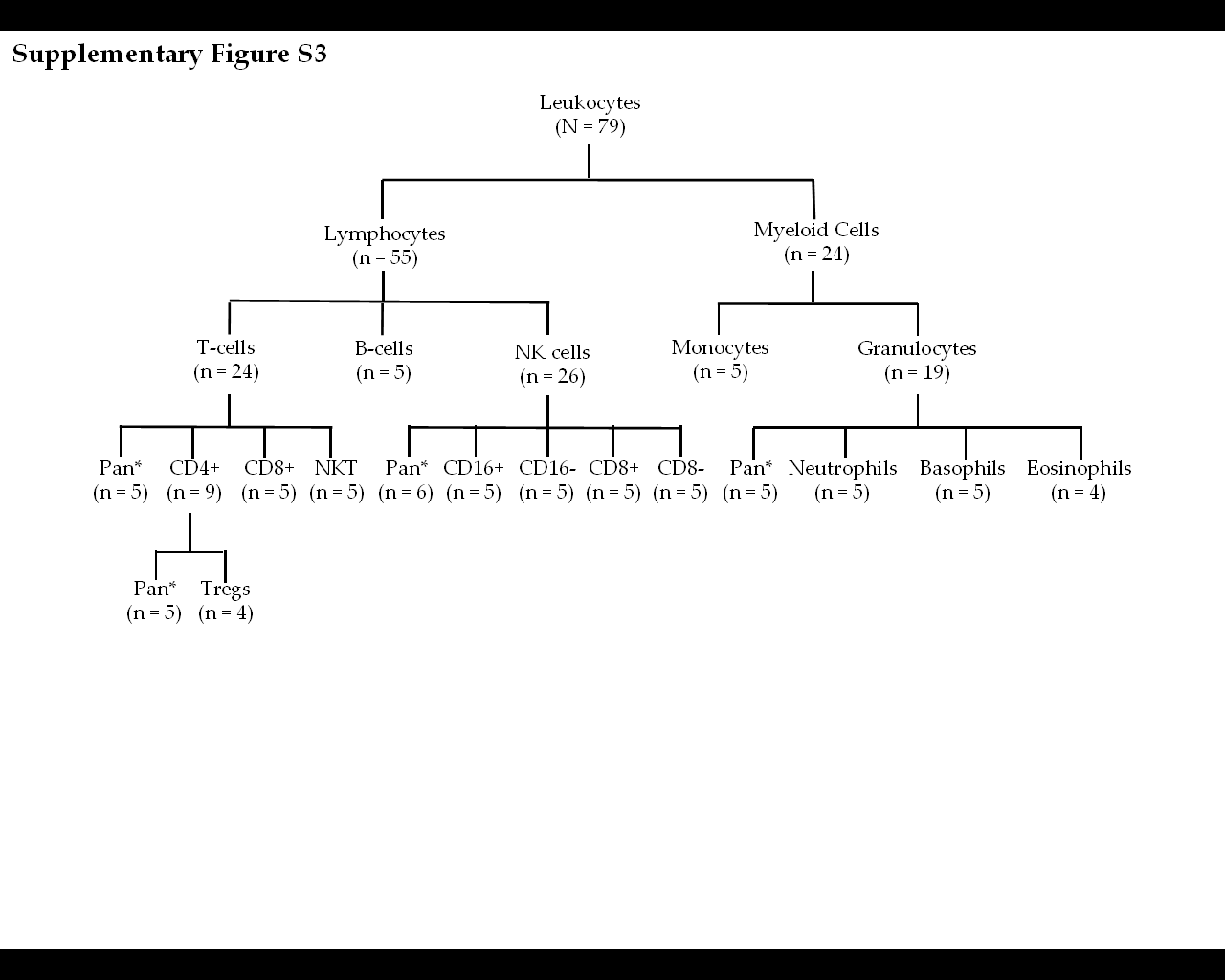


***Additional file 1: Figure S4:*** Heatmap of WBC subset estimates from the high-density DNA methylation microarray. The quantity of each of seven WBC subsets (displayed on the x-axis) was predicted in the purified WBC subset samples, WBC DNA mixtures emulating blood, and human whole blood (WB) samples using DNA methylation. The true identity of each sample is shown on the y-axis, as indicated to the right. Saturation of the interior bins indicate the estimated proportions of WBC subsets, determined using DNA methylation, the samples, as shown in the key at the bottom right.

***
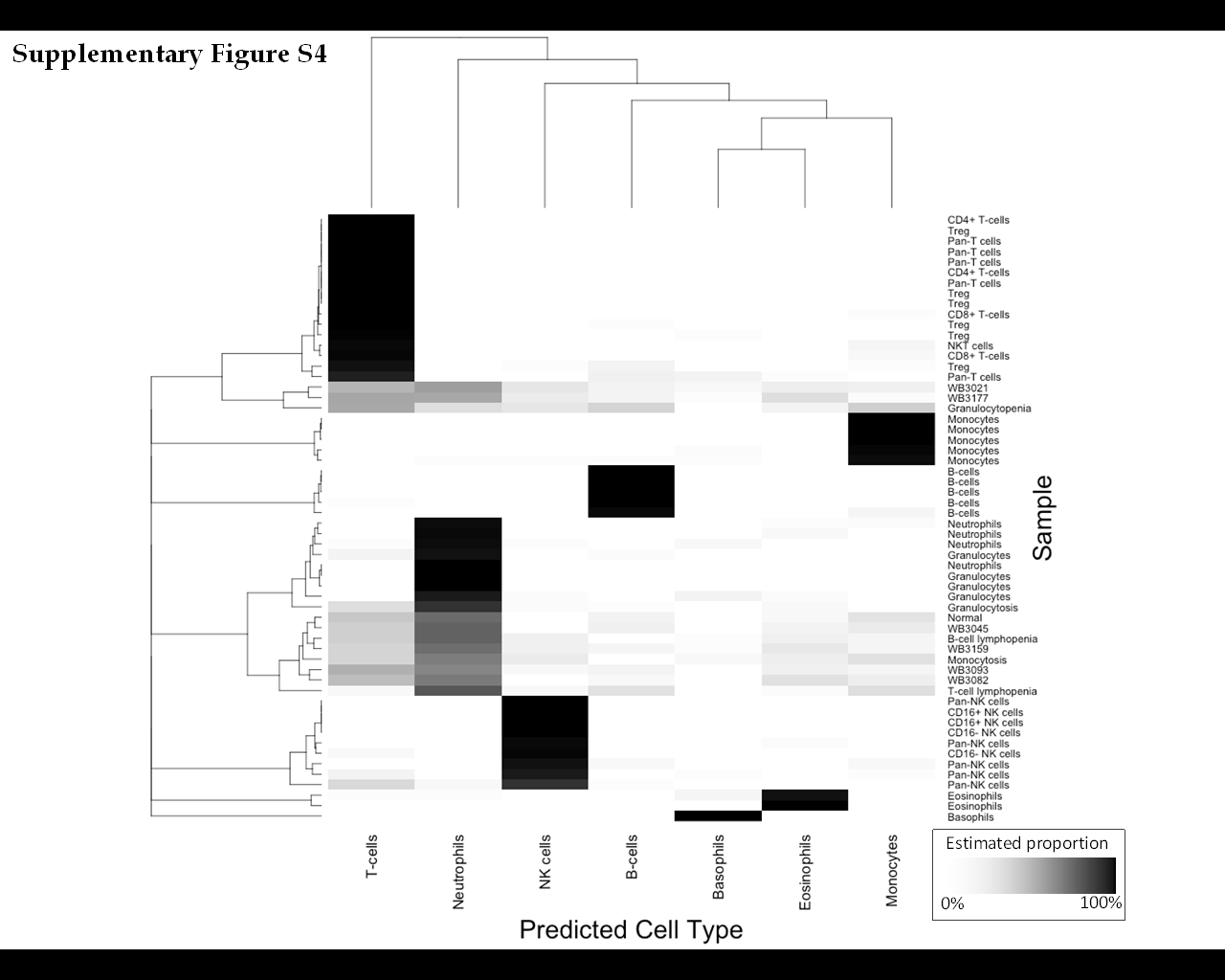
***

***Additional file 1: Figure S5:*** Heatmap of WBC subset estimates from the custom, low-density DNA methylation microarray. The quantity of each of seven WBC subsets (displayed on the x-axis) was predicted in the purified WBC subset samples, WBC DNA mixtures emulating blood, and human whole blood (WB) samples using DNA methylation. The true identity of each sample is shown on the y-axis, as indicated to the right. Saturation of the interior bins indicates the estimated proportions of WBC subsets, determined using DNA methylation, in the samples, as shown in the key at the bottom right.


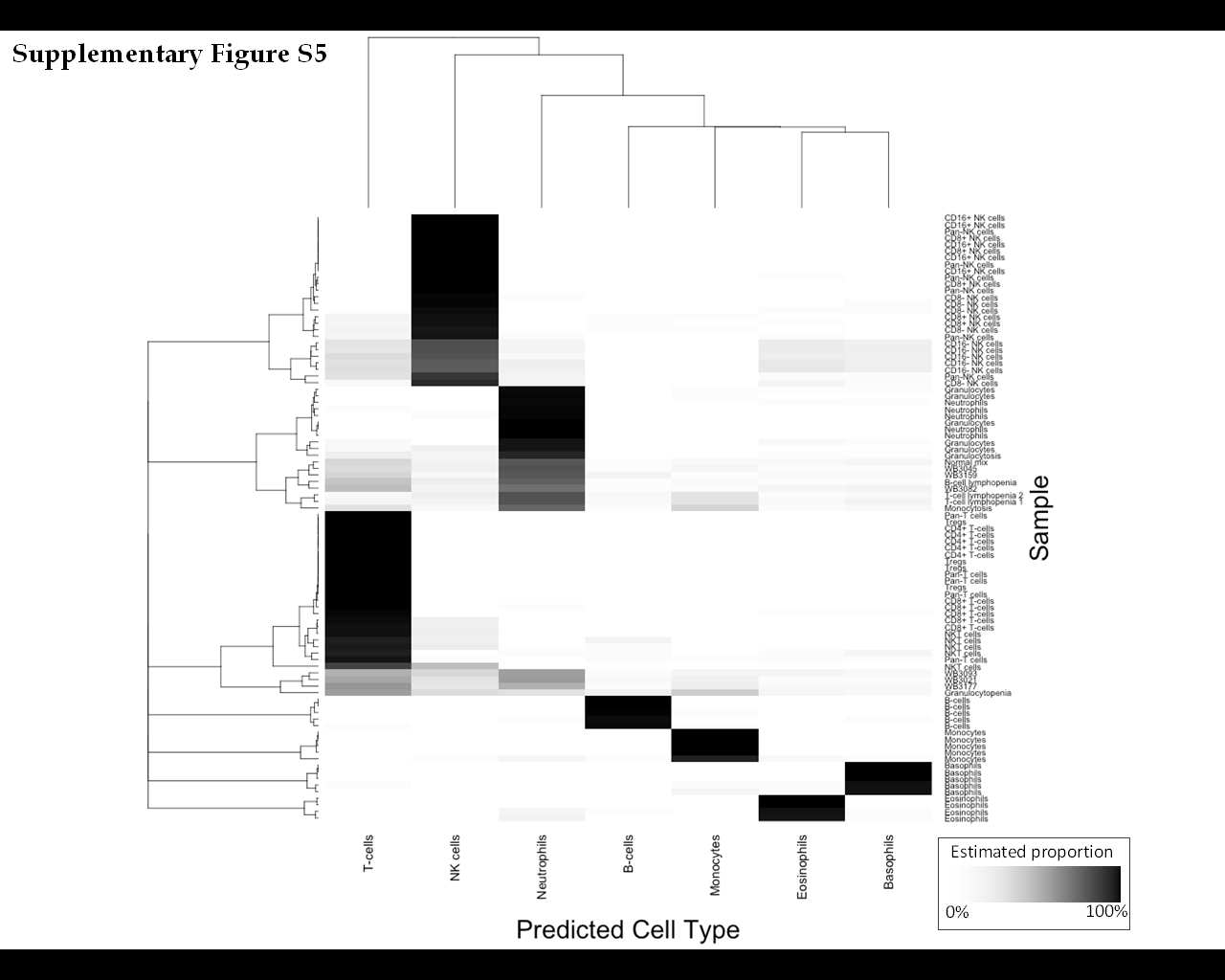


***Additional file 1: Figure S6:*** Quantitative reconstruction of leukocyte subsets using a high-density DNA methylation microarray. In all panels, the x-axis displays quantities of specific WBC subsets determined using DNA methylation. Cell type is indicated by color and sample type is indicated by shape of the point, as described in the inset legends. Lines are drawn from the origin with a slope of one indicating ideal correspondence between the displayed values in each panel. A) DNA from purified WBC subsets was combined in quantities mimicking human blood under clinical conditions. The expected quantity of each cell type is indicated by the y-axis. B-D) Whole blood samples from disease-free human donors subjected to WBC subset quantification by established methods. Results of the established methods are shown on the y-axis and include B) manual 5-part differential C), automated 5-part differential and D) FACS.


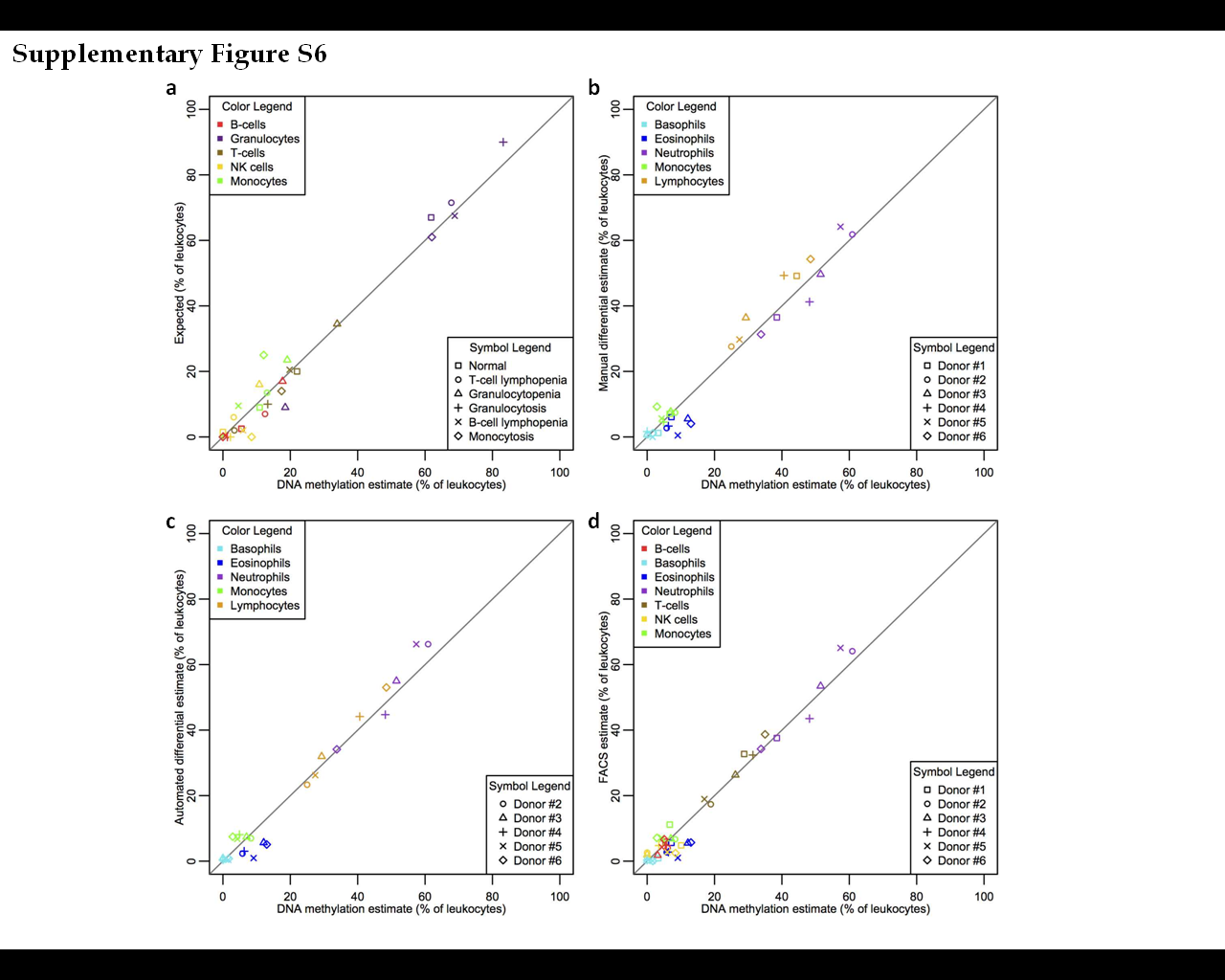


***Additional file 1: Figure S7:*** Comparisons of conventional immune cell quantification methods. In all panels, cell type is indicated by color and disease-free human blood donor is indicated by shape of the point, as described in the legends to the right. Lines are drawn from the origin with a slope of one indicating ideal correspondence between the displayed values in each panel. The conventional methods compared include A) manual 5-part differential and CBC with automated 5-part differential, B) manual 5-part differential and FACS, and B) CBC with automated 5-part differential and FACS.

***
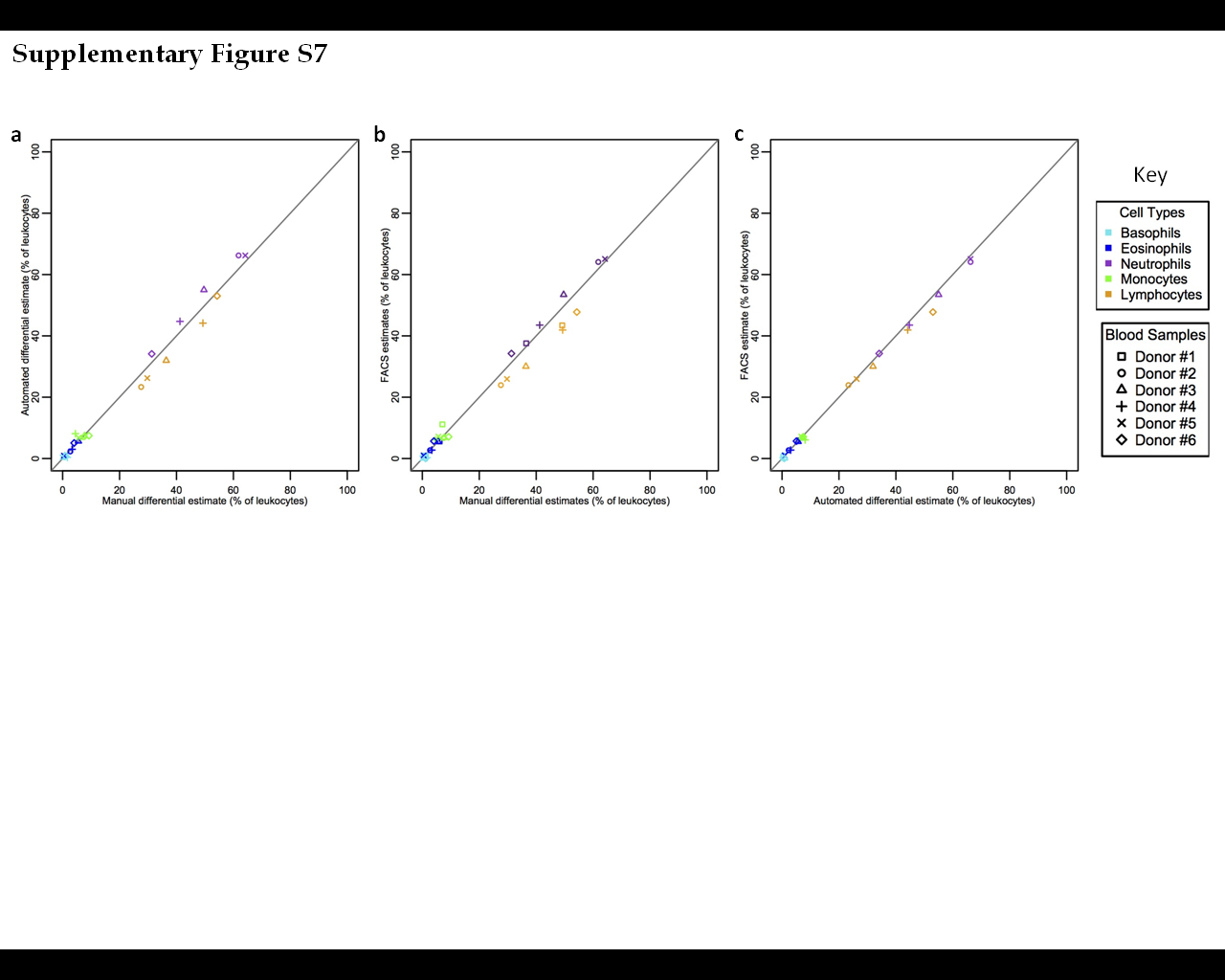
***

***Additional file 1: Figure S8:*** Bland-Altman agreement of expected and observed values. In all panels, each point corresponds to one WBC subset in one sample. The x-axis shows the mean of the given two values of WBC subset quantity (percent) and the y-axis shows the difference between the two values of WBC subset quantities (percent). The root-mean-square-error (RMSE) value between the two values is shown at the top left, in units of WBC subset quantity (percent). Panel A indicates agreement between known amounts of each cell type in laboratory constructed DNA mixtures and measurements obtained using DNA methylation data from the LDMA. Panels B-D indicate agreement between immune cell quantification using DNA methylation (DNAm) from the custom, low-density DNA methylation microarray and B) manual 5-part differential, C) CBC with automated 5-part differential, and D) FACS.


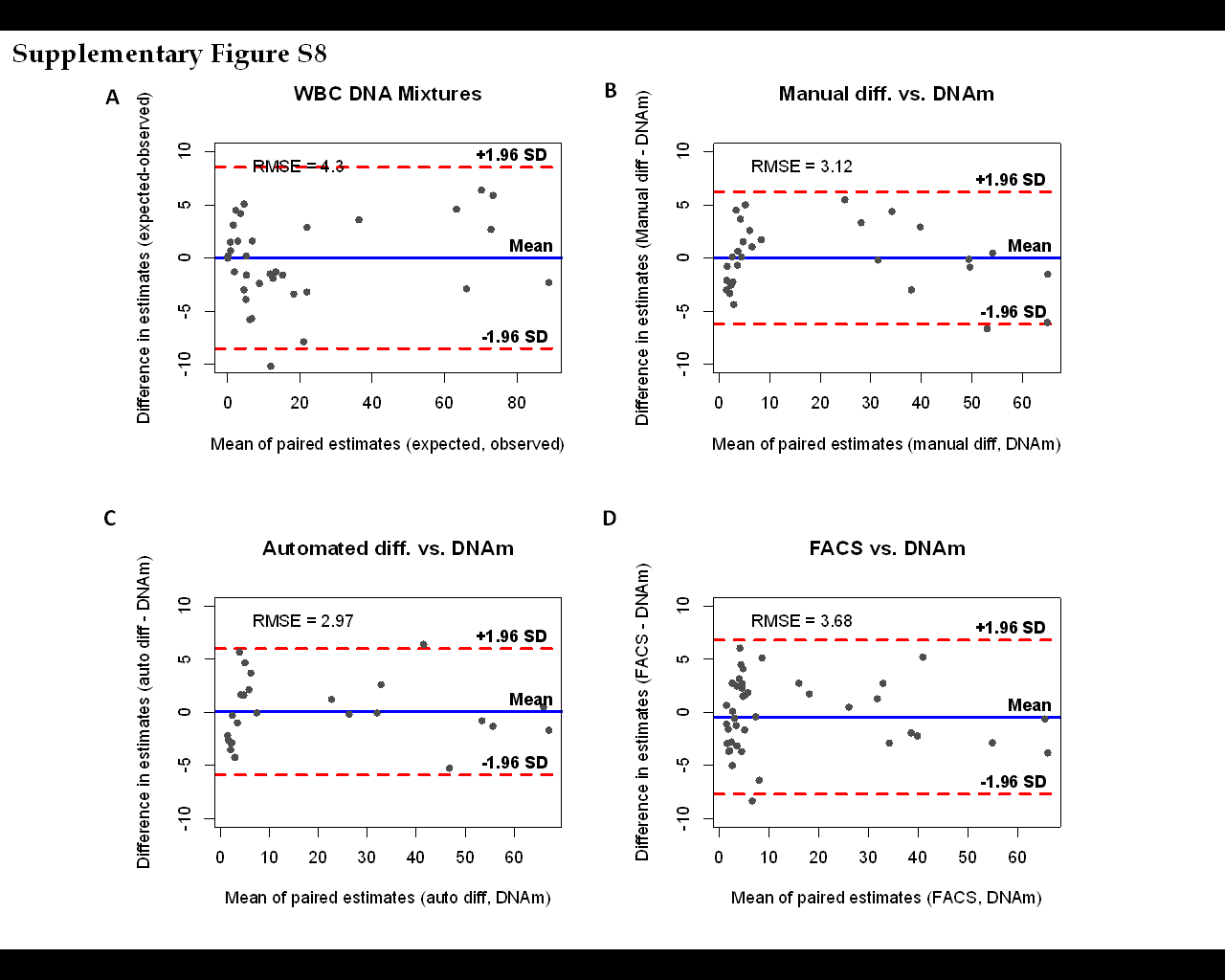


***Additional file 1: Figure S9:*** Bland-Altman agreement of the gold standards. In all panels, each point corresponds to one WBC subset in one blood sample. The x-axis shows the mean of the given two values of WBC subset quantity (percent) and the y-axis shows the difference between the two values of WBC subset quantities (percent). The root-mean-square-error (RMSE) value between the two values is shown at the top left, in units of WBC subset quantity (percent). Panels A-C indicate agreement between conventional immune cell quantification methods, including D) CBC with automated 5-part differential and FACS, E) manual 5-part differential and FACS, and F) manual 5-part differential and CBC with automated 5-part differential.

***
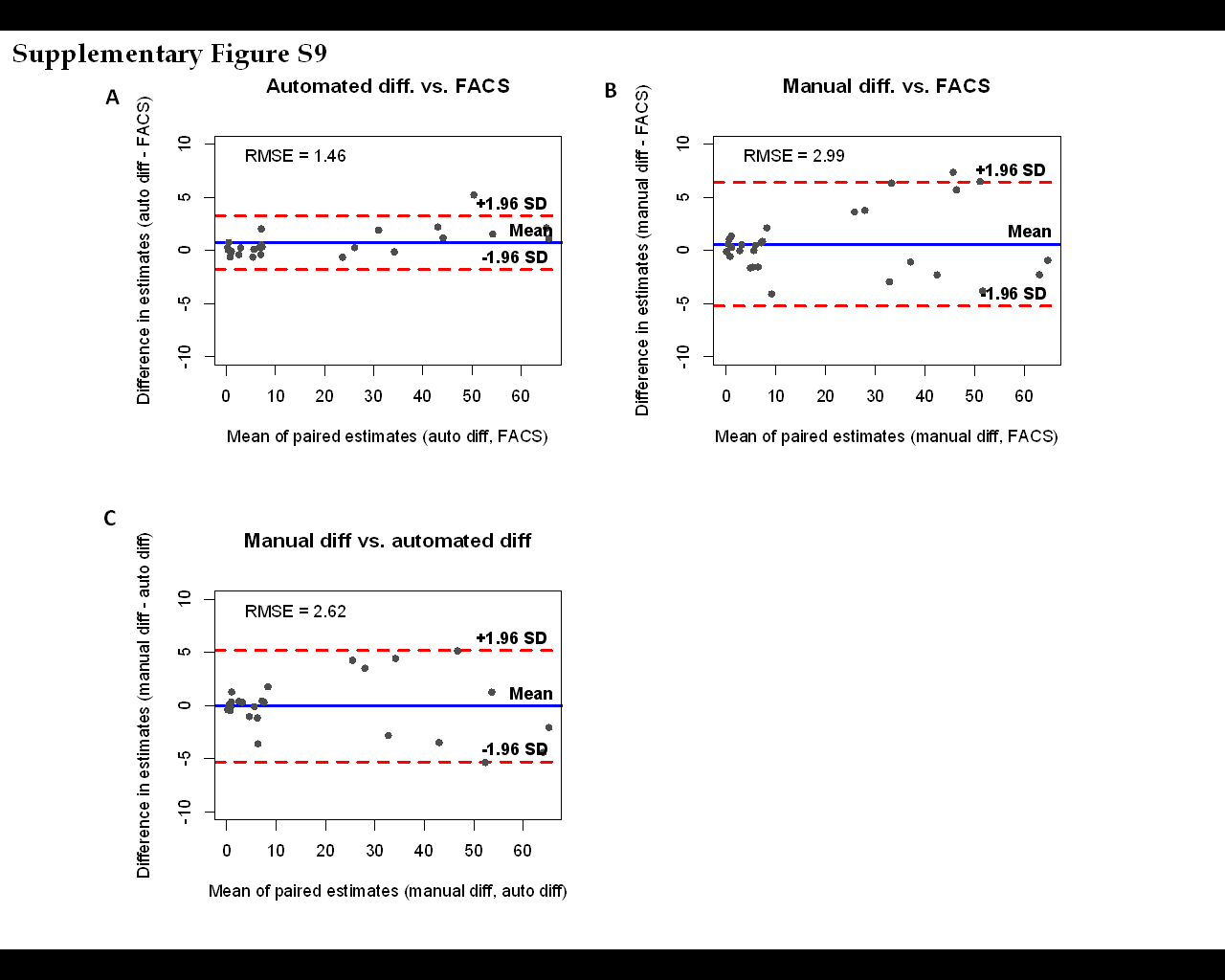
***

***Additional file 1: Figure S10:*** Details of workflow followed for whole blood samples from disease-free human donors that were subjected to established methods of WBC subset quantification as well as quantitative reconstruction of WBC subsets using DNA methylation.


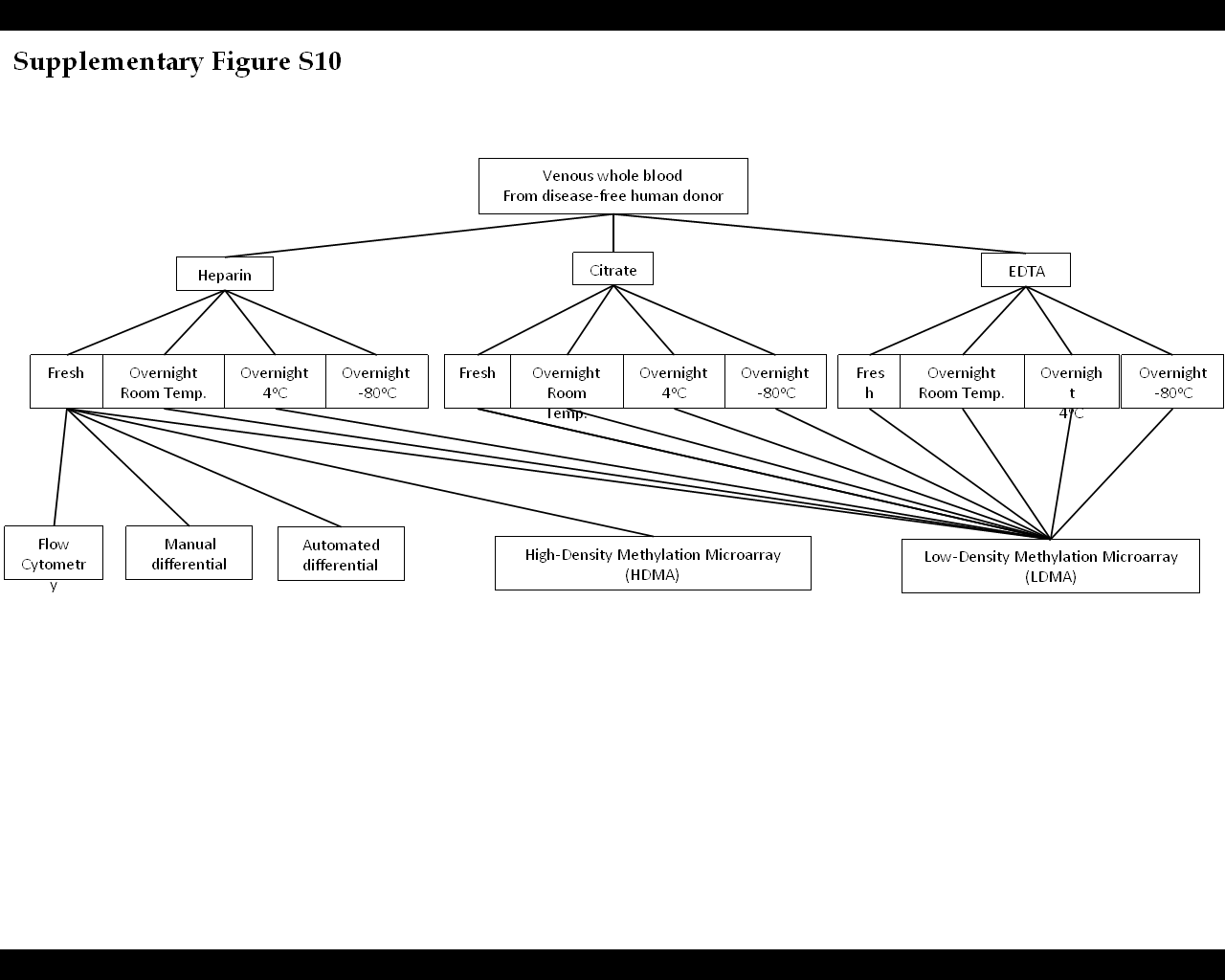

Supplement: Additional file 1 — All supplementary figures referenced in this manuscript. [file gb-2014-15-3-r50-S1.docx]
