# Supplementary material for: Quantitative reconstruction of leukocyte subsets using DNA methylation
Source: Genome Biol. 2014 Mar 5;15(3):R50. doi: 10.1186/gb-2014-15-3-r50 (PMC4053693; doi:10.1186/gb-2014-15-3-r50)
Supplement: Additional file 3: Table S2 — Which displays the demographic characteristics of the six disease-free human donors who provided whole blood samples for comparison to established methods of cell quantification and consideration of blood storage conditions. [file gb-2014-15-3-r50-S3.docx]

| **Supplementary Table S2:** Demographic characteristics of blood donors for comparison to established WBC subset quantification methods | |
| --- | --- |
| **Total**, No. | 6 |
| **Age**, Mean (SD) | 33 (11) |
| **Gender** |  |
| **Male**, No. (%) | 5 (83%) |
| **Female**, No. (%) | 1 (17%) |
| **Race** |  |
| **White**, No. (%) | 6 (100%) |
